# Outcomes of Risk-Stratified Face-to-Face and Teledermoscopy Pathways for Skin Lesion Referrals: A Retrospective Service Evaluation

**DOI:** 10.7759/cureus.113335

**Published:** 2026-07-24

**Authors:** Dalia Eid, Sirag Elaribi, Hannah Ali, Ausama Atwan

**Affiliations:** 1 Department of Dermatology, Aneurin Bevan University Health Board, Newport, GBR; 2 Department of Dermatology, University Hospital Southampton NHS Foundation Trust, Southampton, GBR

**Keywords:** comparative study, face-to-face consultation, skin cancers, teledermatology, telemedicine

## Abstract

Background: Skin cancer referrals to secondary care continue to rise, and teledermoscopy has been incorporated into NHS urgent suspected cancer (USC) pathways to support triage. Direct comparisons of outcomes across deliberately risk-stratified face-to-face (FTF) and teledermoscopy referral pathways, within the same service, time period, and assessing consultant, remain limited.

Methods: We retrospectively compared outcomes across two risk-stratified referral pathways within the same dermatology service over a six-month period (July-December 2025): 201 patients with 207 index lesions assessed via FTF clinics, and 378 patients with 415 lesions assessed via teledermoscopy. All patients were reviewed by the same dermoscopy-trained consultant. Referral urgency, initial clinical impression, clinic outcome, and histology were recorded. The two pre-specified headline outcomes - discharge rate and malignancy yield - are reported as absolute differences between pathways with 95% confidence intervals; all other between-cohort comparisons are descriptive/exploratory and were not adjusted for multiplicity.

Results: FTF referrals were predominantly USC referrals (175/201, 87.0%), with a malignancy yield of 57/207 index lesions (27.5%). The discharge rate was 72/201 patients (35.8%), and 116/201 patients (57.7%) required minor operative procedures. Teledermoscopy referrals were more evenly distributed across urgency categories, with 193/378 patients (51.1%) referred as USC. Malignancy yield, excluding in situ disease, was 60/415 lesions (14.5%), 13.1 percentage points lower than FTF (95% CI: 6.1-20.0); including in situ disease, yield was 63/415 (15.2%), a difference of 13.8 percentage points (95% CI: 6.7-20.9) The discharge rate at initial assessment was 206/378 patients (54.5%), 18.7 percentage points higher than FTF (95% CI: 10.4-27.0). Basal cell carcinoma was the leading confirmed malignancy in both pathways. Incidental lesions were identified in 25/201 FTF assessments (12.4%), of which 24/25 (96.0%) were skin cancers, predominantly basal cell carcinomas.

Conclusions: Within the same service and assessing consultant, teledermoscopy was associated with a substantially higher discharge rate while still detecting skin cancers across referral categories. Much of this difference likely reflects image-based triage allocating lower-suspicion referrals to teledermoscopy, rather than a difference in diagnostic performance between the two modalities; these findings should be interpreted as a descriptive evaluation of pathway throughput rather than a head-to-head comparison of diagnostic accuracy. These findings support the continued, risk-stratified integration of teledermoscopy into NHS skin cancer referral pathways.

## Introduction

Skin cancer is now the most common malignancy diagnosed in England. Incidence rose from 177,677 cases in 2013 to 224,092 in 2019, representing a 26.1% increase driven by an ageing population, cumulative ultraviolet exposure, and improved cancer registration [1]. General practitioners in England and Wales manage more than 13 million dermatology consultations each year, generating approximately one million referrals annually. Around half of these are urgent suspected cancer (USC) referrals, most of which ultimately prove benign [2]. This gap between referral volume and confirmed malignancy reflects the growing pressure on secondary care dermatology services.

Teledermoscopy offers one practical response. The store-and-forward (SAF) model combines high-quality clinical and dermoscopic images with specialist review, enabling triage, identification of suspicious lesions, and discharge of benign cases without a face-to-face (FTF) appointment [3,4]. A Cochrane review of seven studies including 1,588 lesions reported a sensitivity of 94.9% and specificity of 84.3% for detecting skin cancer via teledermatology, broadly comparable with in-person assessment [5]. A separate review focused on non-melanoma skin cancer reached similar conclusions when image quality and clinical history were adequate [6]. Evidence for melanoma diagnosis is directionally consistent, although limited by melanoma's relative rarity within referral cohorts [7]. NHS England formalised this approach in 2022 by incorporating teledermoscopy into the two-week-wait (2WW) pathway [2]; the present evaluation, conducted within NHS Wales, reflects an independently established Welsh service [8,9] rather than the NHS England pathway itself.

Our group has previously shown the Aneurin Bevan University Health Board (ABUHB) teledermoscopy service in Newport to be capacity-releasing, cost-effective, accurate, and well-received by patients [8,9]. A retrospective review of 1,203 referrals also confirmed high outpatient diversion across all urgency categories [3]. However, that earlier work could not directly compare teledermoscopy with FTF assessment within the same service and time frame. Therefore, apparent differences in malignancy yield or clinical disposition may have reflected referral population or clinician variation rather than modality.

This study addresses that gap by comparing FTF and teledermoscopy outcomes within the same service over an identical six-month period, with both pathways assessed by the same dermoscopy-trained consultant. We aimed to describe referral characteristics, clinical disposition, and histologically confirmed malignancy yield between the two modalities.

## Materials and methods

Study design

This retrospective analysis compared teledermoscopy and FTF clinic outcomes for skin lesion referrals between July and December 2025, comprising 201 patients seen FTF and 378 patients seen via teledermoscopy. Referrals are triaged to one of the two pathways by one of 12 consultant dermatologists (including the assessing consultant for this evaluation), with FTF review prioritised for referrals with high-risk or clinically concerning lesions, frail or high-risk patients requiring full-body skin examination, and referrals with images considered too limited for confident remote assessment. Prior to application of exclusion criteria, 401 patients were referred via teledermoscopy and 262 via FTF clinic during the study period; the final analysed cohorts of 378 and 201 patients, respectively, reflect exclusions listed above. Inclusion criteria were new referrals from primary and secondary care with a detailed clinical history and lesion description, accompanied by clinical and dermoscopic images (for teledermoscopy), where the lesion had not previously been assessed by a consultant dermatologist. Exclusion criteria were referrals lacking adequate history or images, cases already reviewed by another consultant dermatologist, and follow-up appointments. Both pathways were reviewed by the same consultant dermatologist with specialist expertise in dermoscopy and skin cancer. Initial clinical impression, confirmed histology, clinic outcome, patient initials, hospital number, and referral urgency were recorded.

Data collection and analysis

Data were extracted from the electronic records of both clinics. Each referral was categorised as USC, urgent, or routine, according to the referring clinician's referral priority. Initial clinical impression was classified as cancer (basal cell carcinoma (BCC), squamous cell carcinoma (SCC), and invasive melanoma), cancer in situ (Bowen's disease/SCC in situ and lentigo maligna (LM)/melanoma in situ), or non-cancer (e.g., actinic keratosis, seborrhoeic keratosis, benign naevi, and other benign lesions). Histology was recorded for all biopsied or excised lesions. For malignancy yield reporting, confirmed histology was classified into five categories reported separately and consistently: BCC, invasive melanoma, invasive SCC, LM/melanoma in situ, and Bowen's disease/SCC in situ. Overall malignancy yield is reported both excluding and including in situ disease (LM and Bowen's disease) as a sensitivity analysis. Clinic outcomes ranged from direct discharge and outpatient follow-up to minor operative procedures (MOPs), referral to other surgical specialties, patient-initiated follow-up (PIFU), further investigation, or repeat photography (digital monitoring). Incidental lesions identified during FTF assessment were recorded separately to allow distinction between index-lesion malignancy yield and additional malignancy detection through wider skin examination.

To ensure methodological accuracy, baseline patient characteristics and clinical outcomes were analysed using the patient as the primary unit of analysis, while initial clinical diagnoses, referral-category distributions, and histological confirmation rates were analysed using the lesion as the primary unit of analysis. Referral urgency and clinical outcome are properties of the referral/patient and were assigned once per patient; where a patient contributed more than one lesion (6/201 FTF, 37/378 teledermoscopy), all of that patient's lesions share the same referral-category and outcome assignment. Diagnostic category and histological confirmation were assessed independently for each lesion. These two units of analysis - patient-level for referral urgency and clinical outcome, lesion-level for diagnostic variables - are used consistently throughout.

Because pathway allocation was based on prior clinical and image-based triage rather than randomisation, between-cohort comparisons do not estimate an independent effect of assessment modality. All between-cohort analyses are therefore reported descriptively, using absolute differences with 95% confidence intervals for the two pre-specified headline outcomes (discharge rate, malignancy yield). Remaining comparisons are exploratory, unadjusted for multiplicity, and should not be interpreted as confirmatory.

Most data are presented descriptively as numbers and percentages (n, %). To compare the overall distribution of clinical outcomes, initial diagnoses, and referral categories between the FTF and teledermoscopy cohorts, Pearson's chi-square (χ²) tests of independence were performed using observed counts. To localize specific differences between the pathways, post-hoc row-wise χ² comparisons were executed for individual diagnostic, outcome, and referral categories against all other combined categories. For 2 x 2 comparisons with an expected cell count below five, Fisher's exact test was used and the corresponding odds ratio (OR) reported; for larger contingency tables (e.g., 2 × 3 referral-category comparisons) with an expected cell count below five, the Freeman-Halton extension of Fisher's exact test was used instead, for which an odds ratio is not applicable. Baseline demographic characteristics, including sex and age, were also compared between cohorts. Sex distribution was compared using Pearson's chi-square test. Age was non-normally distributed in both cohorts, with a Shapiro-Wilk p < 0.001, and was therefore compared between cohorts using the Mann-Whitney U test. Age is reported as mean (SD) and median (IQR). Statistical significance across all tests was set at p < 0.05.

As this retrospective study represents a service evaluation of routinely collected data, it was reviewed by the ABUHB Research and Development Department, which confirmed that formal ethical approval was not required, in keeping with institutional policy; no separate registration number was assigned. Identifiable data (patient initials, hospital numbers) were used only to link clinic records during extraction, were stored on a secure NHS Wales-approved OneDrive account with restricted access, and were removed prior to statistical analysis.

## Results

Patients demographics

Baseline demographics differed between the two cohorts (Table 1). The FTF cohort comprised 201 patients: 93 female (46.3%) and 108 male (53.7%), with a mean age of 73.71 years (SD: 15.07; median: 77 years, IQR: 68-84). The teledermoscopy cohort comprised 378 patients: 222 female (58.7%) and 156 male (41.3%), with a mean age of 59.56 years (SD: 17.36; median: 63 years, IQR: 49-72). Sex distribution differed significantly between cohorts (χ²(1) = 8.21, p = 0.004), with a higher proportion of male patients assessed FTF. The FTF cohort was also significantly older than the teledermoscopy cohort (Mann-Whitney U = 56862, p < 0.0001). This likely reflects the triage of older, higher-risk patients and those with more clinically suspicious lesions, directly to FTF review.

**Table 1:** Patient demographics for the face-to-face and teledermoscopy cohorts Data are presented as number (%) for categorical variables and as mean (SD)/median (IQR) for age (patient as the unit of analysis, n=201 face-to-face, n=378 teledermoscopy). Sex distribution was compared using Pearson's chi-square test of independence. Age in both cohorts was non-normally distributed (Shapiro-Wilk p<0.001), consistent with the right-skew indicated by the gap between mean and median; age was therefore compared between cohorts using the Mann-Whitney U test rather than an independent-samples t-test.

Demographic	Face-to-face cohort, n=201	Teledermoscopy cohort, n=378	Test statistic	p-value
Female, n (%)	93 (46.3%)	222 (58.7%)	χ²(1) = 8.21	0.004
Male, n (%)	108 (53.7%)	156 (41.3%)		
Mean age, years (SD)	73.71 (15.07)	59.56 (17.36)	U = 56862	<0.0001
Median age, years (IQR)	77 (68-84)	63 (49-72)		

FTF-assessed cohort

Of the 201 patients assessed FTF, there were 207 index lesions, excluding incidental lesions. In total, 175 patients (87.0%) were USC referrals, 18 (9.0%) were urgent, and 8 (4.0%) were routine. A further 25 incidental lesions were identified during FTF assessment.

Clinically, 116 (57.7%) patients were booked for MOPs, while 72 (35.8%) were discharged following review. Smaller proportions were referred to another specialty (6, 3.0%); given an outpatient follow-up appointment (4, 2.0%); offered PIFU (2, 1.0%); or referred for further investigation with ultrasound (1, 0.5%).

**Figure 1:**
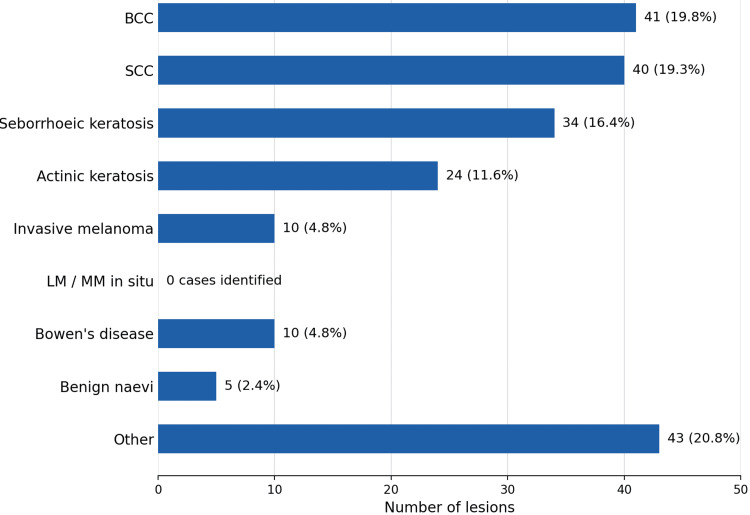
Initial clinical diagnoses Initial clinical diagnoses among face-to-face assessed index lesions (n=207). Bars show the number and percentage of lesions assigned to each diagnostic category at initial clinical assessment, prior to histological confirmation. No lentigo maligna/melanoma in situ cases were identified at initial assessment in the face-to-face cohort. "Other" comprises all remaining diagnostic categories not individually listed. BCC: basal cell carcinoma; SCC: squamous cell carcinoma; LM: lentigo maligna

Initial clinical diagnoses were mainly keratinocyte cancers and premalignant lesions (Figure 1): BCC of 41/207 (19.8%), SCC of 40/207 (19.3%), seborrhoeic keratosis of 34/207 (16.4%), actinic keratosis of 24/207 (11.6%), invasive melanoma of 10/207 (4.8%), 0 suspected lentigo maligna/melanoma in situ, Bowen's disease of 10/207 (4.8%), and benign naevi of 5/207 (2.4%). The remaining 43 lesions (20.8%) fell into other diagnostic categories.

**Figure 2:**
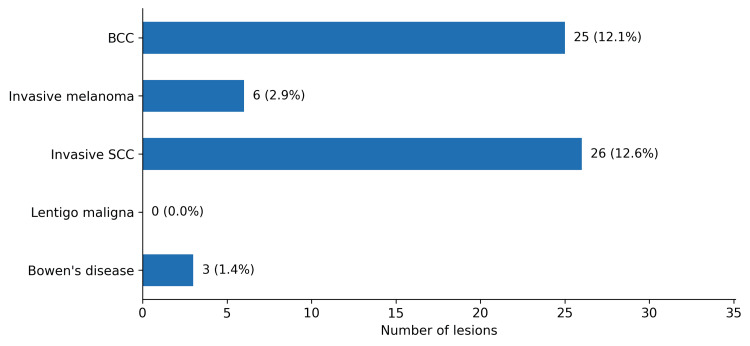
Histology-confirmed malignant and pre-malignant lesions Histology-confirmed malignant and pre-malignant lesions among face-to-face assessed index lesions (n=207). Bars show the number and percentage of lesions confirmed malignant or pre-malignant on histology for basal cell carcinoma (BCC), squamous cell carcinoma (SCC), invasive melanoma, lentigo maligna/melanoma in situ, and Bowen's disease/SCC in situ.

Histology confirmed 25 BCCs (12.1%), 6 invasive melanomas (2.9%), 26 invasive SCCs (12.6%), 0 lentigo maligna (0%), and 3 Bowen's disease (1.4%) among the 207 index lesions. This gives a malignancy yield of 57/207 (27.5%) excluding in situ disease, or 60/207 (29.0%) including in situ disease (Figure 2).

**Figure 3:**
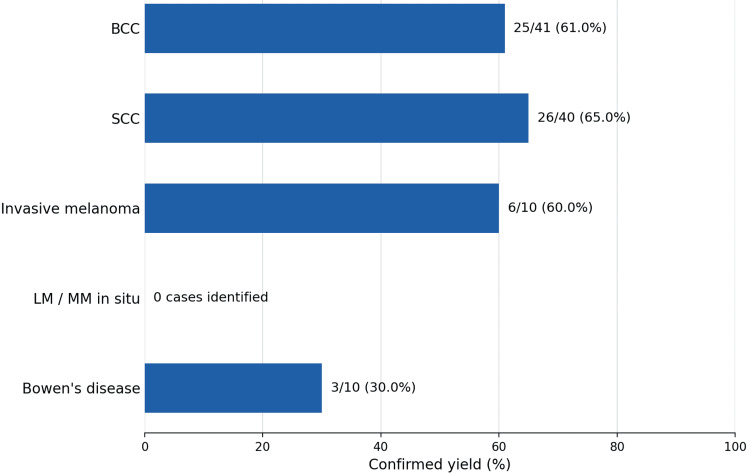
Histological confirmation yield among clinically suspected lesions (face-to-face cohort) Proportion (n/N, %) of clinically suspected lesions in each diagnostic category subsequently confirmed by histology, among face-to-face-assessed lesions (BCC, SCC, invasive melanoma, lentigo maligna/melanoma in situ, and Bowen's disease). No lentigo maligna/melanoma in situ cases were identified in the face-to-face cohort. N is the total number of clinically suspected lesions in each category, including those not sent for biopsy; this therefore represents a confirmation yield rather than diagnostic accuracy or positive predictive value (see Limitations) BCC: basal cell carcinoma; SCC: squamous cell carcinoma; LM: lentigo maligna

Confirmation yield was broadly similar for suspected BCC, SCC, and invasive melanoma, ranging from 60.0-65.0% (Figure 3). No lentigo maligna/melanoma in situ cases were identified in the FTF cohort. Confirmation yield was lower for Bowen's disease (3/10, 30.0%).

**Figure 4:**
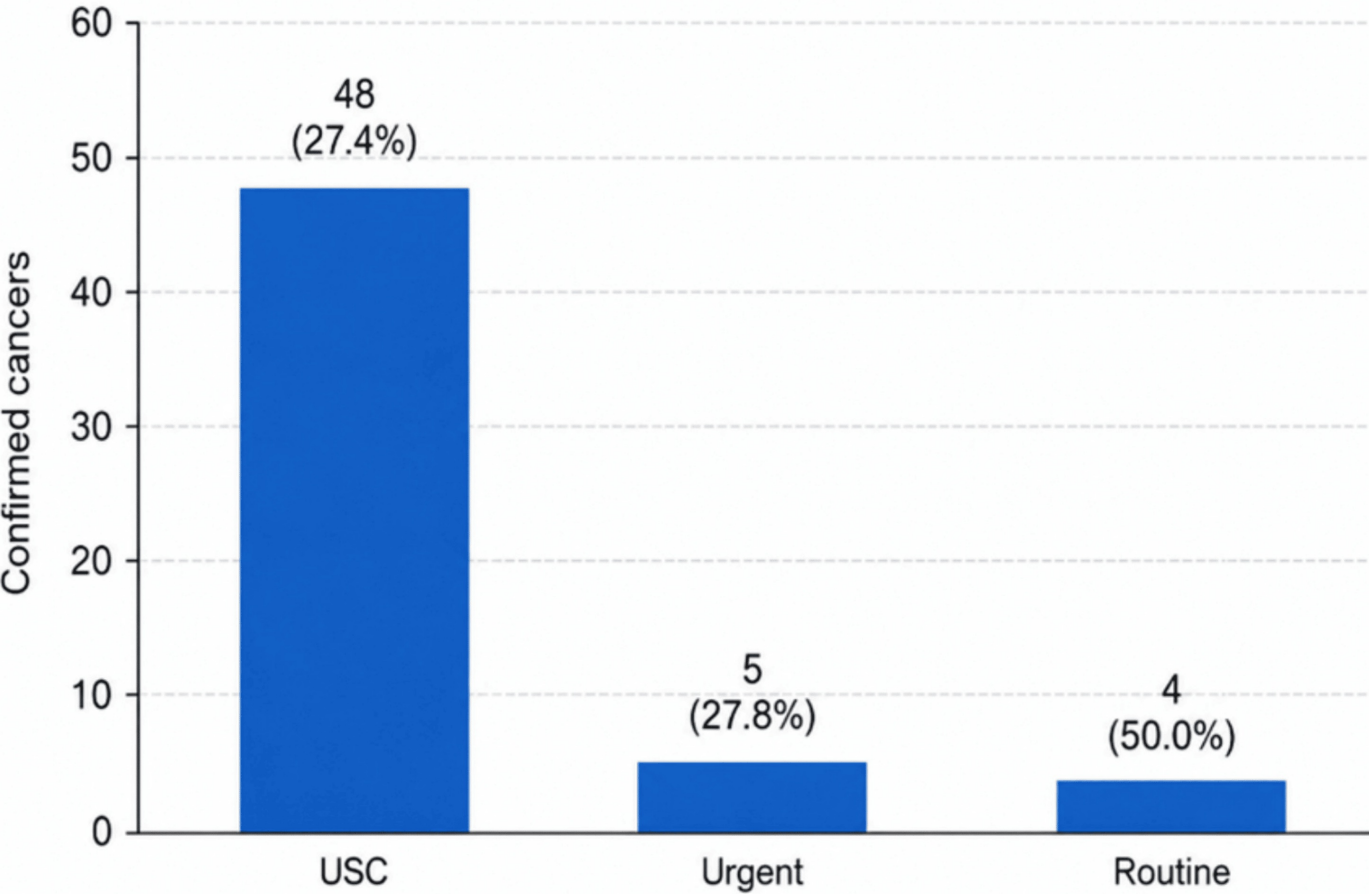
Confirmed cancers by referral category Histology-confirmed cancers by referral category among face-to-face-assessed patients. Bars show the number and percentage of confirmed cancers (BCC, SCC, and melanoma/lentigo maligna combined) within each referral urgency group: urgent suspicion of cancer (USC), urgent, and routine. Referral category is assigned per patient/referral; confirmed cancer counts are per lesion (see Methods).

By referral category (Figure 4), confirmed cancers were concentrated in the USC group, comprising 20 BCCs, 22 SCCs, and 6 invasive melanomas, giving a total malignancy yield of 48/175 (27.4%). The urgent group yielded 5/18 confirmed cancers (27.8%), while the routine group yielded 4/8 confirmed cancers (50.0%), although the latter should be interpreted cautiously given the small denominator.

**Figure 5:**
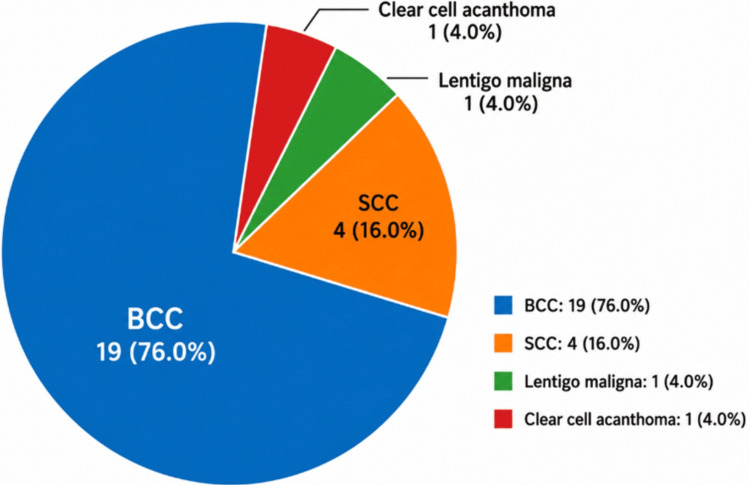
Incidental lesions identified Diagnostic breakdown of incidental lesions identified during face-to-face assessment (n=25). Incidental lesions were identified outside the referred index lesion during full skin examination and are shown by confirmed diagnosis: BCC, SCC, lentigo maligna, and clear cell acanthoma. BCC: basal cell carcinoma; SCC: squamous cell carcinoma

Incidental lesions were an important additional finding (Figure 5). In total, 25 incidental lesions were identified, comprising 19 BCCs (76.0%), 4 SCCs (16.0%), 1 lentigo maligna/melanoma in situ (4.0%), and 1 clear cell acanthoma (4.0%); 24 of the 25 incidental lesions were skin cancers. When incidental malignancies were included, the total number of cancers detected increased from 57 to 81 across 232 assessed lesions, giving an overall cancer detection rate of 34.9%.

Teledermoscopy-assessed cohort

Of the 378 patients assessed via teledermoscopy, there were 415 lesions. In total, 193 patients (51.1%) were USC referrals, 118 (31.2%) were urgent, and 67 (17.7%) were routine, representing a more even distribution than in the FTF cohort.

Clinically, 206 (54.5%) patients were discharged following review, while 74 (19.6%) were given a follow-up outpatient appointment, and 71 (18.8%) were booked for MOPs. The remaining patients underwent repeat photography (16, 4.2%); were referred to another specialty (10, 2.6%); or were offered PIFU (1, 0.3%). Of the 16 patients who underwent repeat photography, 10 (62.5%) were subsequently discharged, 3 (18.8%) required FTF review, and 3 (18.8%) proceeded to MOPs.

Across most diagnostic categories, the largest proportion of referrals came through the USC pathway. For example, USC referrals accounted for 65/116 seborrhoeic keratoses (56.0%), 31/73 BCCs (42.5%), and 17/20 melanoma/lentigo maligna diagnoses (85.0%).

Histology confirmed 54 BCCs (13.0%), 2 invasive melanomas (0.5%), 4 invasive SCCs (1.0%), 1 lentigo maligna/melanoma in situ (0.2%), and 2 Bowen's disease (0.5%) among the 415 lesions. This gives a malignancy yield of 60/415 (14.5%) excluding in situ disease, or 63/415 (15.2%) including in situ disease.

**Figure 6:**
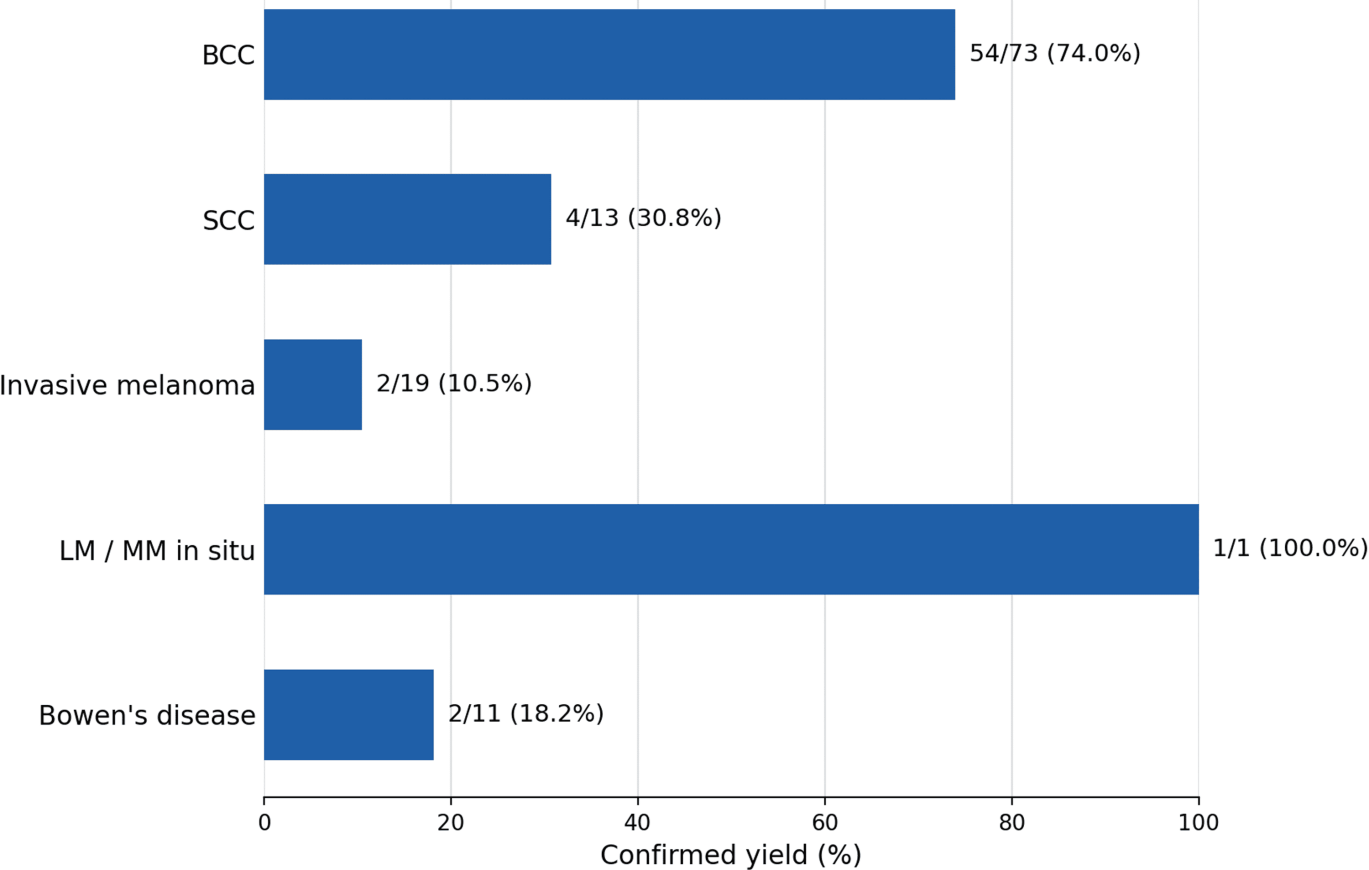
Histological confirmation yield among clinically suspected lesions (teledermoscopy cohort) Proportion (n/N, %) of clinically suspected lesions in each diagnostic category subsequently confirmed by histology, among teledermoscopy-assessed lesions (BCC, SCC, invasive melanoma, lentigo maligna/melanoma in situ, and Bowen's disease). The lentigo maligna/melanoma in situ yield (1/1, 100%) is based on a single case and should not be over-interpreted. N is the total number of clinically suspected lesions in each category, including those not sent for biopsy; this therefore represents a confirmation yield rather than diagnostic accuracy or positive predictive value (see Limitations). BCC: basal cell carcinoma; SCC: squamous cell carcinoma; LM: lentigo maligna

Confirmation yield varied by suspected diagnosis (Figure 6). Suspected BCC had the highest yield at 54/73 (74.0%), followed by SCC at 4/13 (30.8%), invasive melanoma at 2/19 (10.5%), lentigo maligna/melanoma in situ at 1/1 (based on a single case), and Bowen's disease at 2/11 (18.2%). Actinic keratosis, seborrhoeic keratosis, and benign naevi are not routinely biopsied and are therefore not reported as confirmation yields (see Limitations).

**Figure 7:**
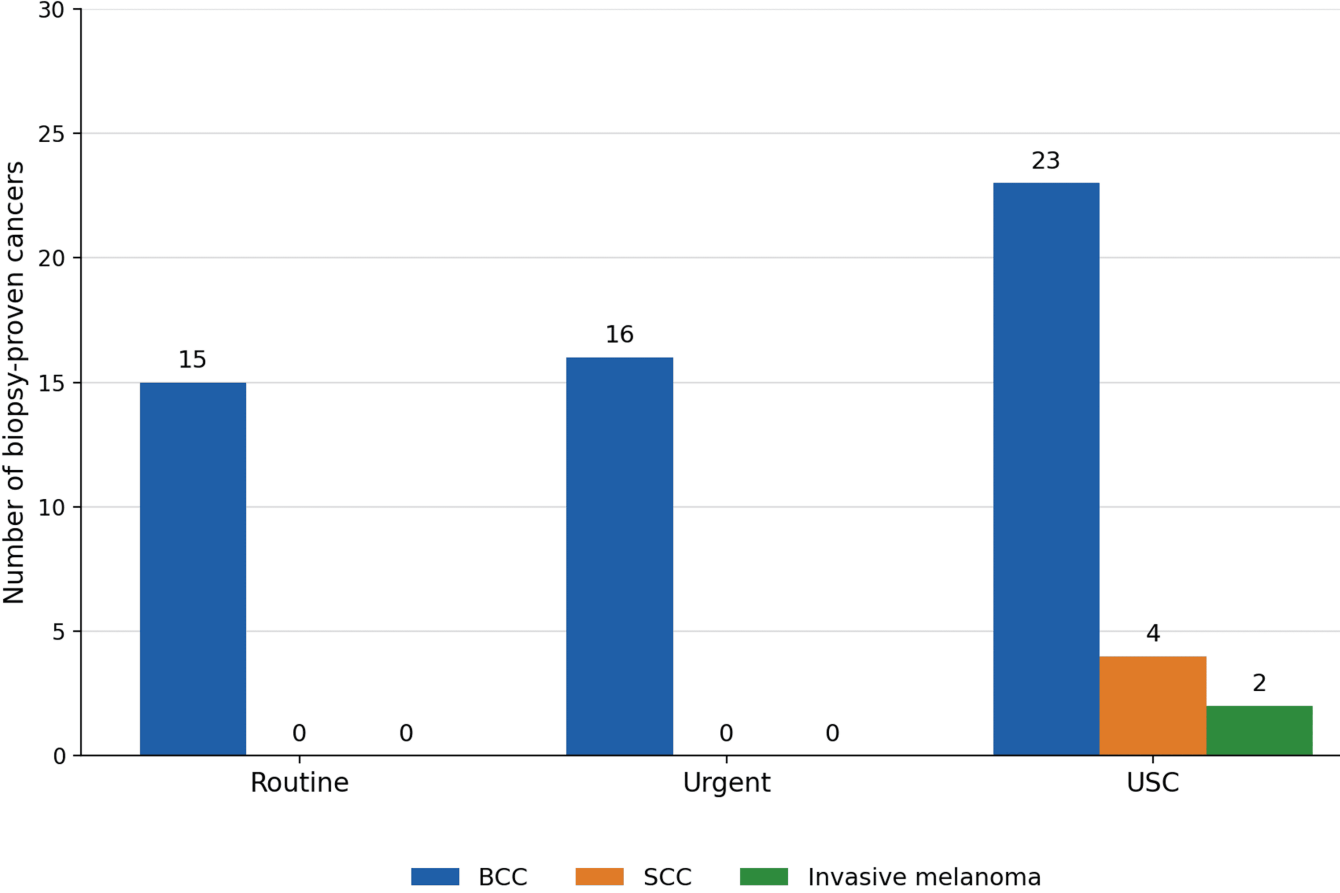
Biopsy-proven cancers by referral group Biopsy-proven cancers by referral category within the teledermoscopy-assessed cohort. Bars show the number of confirmed BCC, SCC, and invasive melanoma cases within each referral urgency group (routine, urgent, USC). SCC and invasive melanoma were confirmed only within the USC group. The single lentigo maligna/melanoma in situ case is excluded from this figure and reported separately (see Table 4). Referral category is assigned per patient/referral; confirmed cancer counts are per lesion (see Methods). BCC: basal cell carcinoma; SCC: squamous cell carcinoma

Biopsy-proven BCC was identified across all referral groups (Figure 7), with the highest proportion seen among routine referrals (15/67, 22.4%). Confirmed SCC (4/193, 2.1%) and invasive melanoma (2/193, 1.0%) were both identified exclusively within the USC group, as was the single confirmed lentigo maligna/melanoma in situ case, which is reported separately and excluded from Figure 7.

Comparison of clinical outcomes and diagnoses between cohorts

The overall distribution of clinical outcomes differed significantly between the FTF and teledermoscopy cohorts (Table 2; χ²(5) = 108.87, p < 0.0001). Teledermoscopy was associated with a substantially higher discharge rate than FTF assessment (206/378, 54.5% vs 72/201, 35.8%; absolute difference: 18.7 percentage points, 95% CI: 10.4-27.0). Unadjusted significance tests for this and other pathway comparisons are retained in Table 2 for completeness but are exploratory given the non-randomised triage design (see Methods). In contrast, FTF assessment was associated with a significantly higher proportion of patients booked or referred for MOPs (116/201, 57.7% vs 71/378, 18.8%; χ²(1) = 90.94, p < 0.0001).

**Table 2:** Comparison of clinical outcomes between face-to-face and teledermoscopy cohorts Data are presented as number (%), n (%). Statistical comparisons were performed using observed counts rather than percentages, as recommended for χ² testing. Row-wise comparisons were performed using Pearson's Chi-square test for each outcome versus all other outcomes. Fisher's exact test was used for PIFU because of the small cell counts, and the odds ratio (OR) is therefore reported instead of a χ² statistic. The overall distribution of clinical outcomes between the face-to-face and teledermoscopy cohorts was compared using Pearson's chi-square test of independence. Row-wise chi-square/Fisher's exact comparisons are exploratory and unadjusted for multiplicity; they should not be interpreted as confirmatory given the non-randomised triage design (see Methods).

Clinical outcome	Face-to-face cohort, n=201	Teledermoscopy cohort, n=378	Test statistic	p-value
Discharged	72 (35.8%)	206 (54.5%)	χ²(1) = 18.34	<0.0001
Booked/referred for MOPs	116 (57.7%)	71 (18.8%)	χ²(1) = 90.94	<0.0001
Referred to other specialties	6 (3.0%)	10 (2.6%)	χ²(1) = 0.06	0.812
Follow-up/OPD follow-up	4 (2.0%)	74 (19.6%)	χ²(1) = 34.82	<0.0001
Patient-initiated follow-up (PIFU)	2 (1.0%)	1 (0.3%)	OR = 3.79	0.278
Further investigation/repeat photography	1 (0.5%)	16 (4.2%)	χ²(1) = 6.42	0.011
Overall clinical outcome distribution	201 (100%)	378 (100%)	χ²(5) = 108.87	<0.0001

Referral to other specialties did not differ significantly between cohorts (χ²(1) = 0.06, p = 0.812), and PIFU rates were also comparable (Fisher's exact test, OR = 3.79, p = 0.278). Follow-up or outpatient review was significantly more frequent in the teledermoscopy cohort (χ²(1) = 34.82, p < 0.0001), as was further investigation or repeat photography (χ²(1) = 6.42, p = 0.011).

The overall distribution of initial clinical diagnoses differed significantly between cohorts (Table 3; χ²(8) = 70.88, p < 0.0001). SCC was significantly more frequent in the FTF cohort than in the teledermoscopy cohort (40/207 (19.3%) vs 13/415 (3.1%); χ²(1) = 46.45, p < 0.0001). In contrast, seborrhoeic keratosis (34/207 (16.4%) vs 116/415 (28.0%); χ²(1) = 10.03, p = 0.002) and benign naevi (5/207 (2.4%) vs 56/415 (13.5%); χ²(1) = 19.16, p < 0.0001) were significantly more frequent in the teledermoscopy cohort. BCC, actinic keratosis, invasive melanoma, lentigo maligna/melanoma in situ, Bowen's disease, and other diagnoses did not differ significantly between cohorts (all p > 0.05). Table 3 presents the comparison of initial clinical diagnoses between face-to-face and teledermoscopy cohorts.

**Table 3:** Comparison of initial clinical diagnoses between face-to-face and teledermoscopy cohorts Data are presented as number (%), n (%), of total lesions in each cohort. Row-wise comparisons were performed using Pearson's Chi-square test for each diagnosis versus all other diagnoses, except lentigo maligna/melanoma in situ, for which Fisher's exact test was used owing to an expected cell count below 5. The overall diagnostic distribution was compared using a 2 × 9 Pearson's chi-square test, including the eight listed diagnoses plus "other diagnoses." P-values are unadjusted for multiple comparisons. Row-wise chi-square/Fisher's exact comparisons are exploratory and unadjusted for multiplicity; they should not be interpreted as confirmatory given the non-randomised triage design (see Methods).

Diagnosis	Face-to-face cohort, n=207	Teledermoscopy cohort, n=415	Test statistic	p-value
Seborrhoeic keratosis	34 (16.4%)	116 (28.0%)	χ²(1) = 10.03	0.002
BCC	41 (19.8%)	73 (17.6%)	χ²(1) = 0.45	0.501
Benign naevi	5 (2.4%)	56 (13.5%)	χ²(1) = 19.16	<0.0001
Actinic keratosis	24 (11.6%)	39 (9.4%)	χ²(1) = 0.73	0.392
Invasive melanoma	10 (4.8%)	19 (4.6%)	χ²(1) = 0.02	0.887
Lentigo maligna/melanoma in situ	0 (0.0%)	1 (0.2%)	Fisher's exact	1
SCC	40 (19.3%)	13 (3.1%)	χ²(1) = 46.45	<0.0001
Bowen's disease	10 (4.8%)	11 (2.7%)	χ²(1) = 2.01	0.156
Other diagnoses	43 (20.8%)	87 (21.0%)	χ²(1) = 0.00	0.956
Overall diagnostic distribution	207 (100%)	415 (100%)	χ²(8) = 70.88	<0.0001

Referral-category distribution differed descriptively between cohorts, most notably for seborrhoeic keratosis and actinic keratosis, consistent with image-based triage directing lower-suspicion referrals to teledermoscopy. Detailed referral-category counts by diagnosis are available from the authors on request.

Comparing biopsy-confirmed diagnoses across the whole cohort (Table 4), BCC was similarly frequent in the FTF and teledermoscopy cohorts (25/207 (12.1%) vs 54/415 (13.0%); absolute difference: 0.9 percentage points, 95% CI: -4.6 to 6.4). Invasive SCC was more frequent in the FTF cohort (26/207 (12.6%) vs 4/415 (1.0%); absolute difference: -11.6 percentage points, 95% CI: -16.2 to -7.0), as was invasive melanoma to a lesser extent (6/207 (2.9%) vs 2/415 (0.5%); absolute difference: -2.4 percentage points, 95% CI: -4.8 to 0.0). In situ disease was uncommon in both cohorts: lentigo maligna was identified in 0/207 FTF lesions vs 1/415 teledermoscopy lesions, and Bowen's disease in 3/207 (1.4%) vs 2/415 (0.5%). These comparisons are exploratory and reported as absolute differences with 95% confidence intervals rather than significance tests, consistent with the descriptive framing adopted throughout (see Methods). Percentages use the total number of assessed index lesions (207 FTF, 415 teledermoscopy) as the denominator, consistent with the lesion-level denominator used for the malignancy yields reported in Figure 2 and in the text above.

**Table 4:** Comparison of biopsy-confirmed diagnostic categories between face-to-face and teledermoscopy cohorts Data are presented as n/N (%) using the total number of assessed index lesions as the denominator (N=207 face-to-face, N=415 teledermoscopy), consistent with the lesion-level denominator used throughout for diagnostic categories (see Methods). Between-cohort comparisons are reported as absolute differences with 95% confidence intervals rather than significance tests, consistent with the descriptive, exploratory framing adopted for all non-headline comparisons in this study (see Methods and Limitations). Malignancy yield is reported both excluding and including in situ disease (lentigo maligna and Bowen's disease) as a sensitivity analysis (see Discussion). BCC: basal cell carcinoma; SCC: squamous cell carcinoma

Category	Face-to-face, n/N (%)	Teledermoscopy, n/N (%)	Absolute difference (95% CI)
BCC	25/207 (12.1%)	54/415 (13.0%)	0.9pp (−4.6 to 6.4)
Invasive melanoma	6/207 (2.9%)	2/415 (0.5%)	−2.4pp (−4.8 to 0.0)
Invasive SCC	26/207 (12.6%)	4/415 (1.0%)	−11.6pp (−16.2 to −7.0)
Lentigo maligna (melanoma in situ)	0/207 (0.0%)	1/415 (0.2%)	0.2pp (−0.2 to 0.7)
Bowen's disease (SCC in situ)	3/207 (1.4%)	2/415 (0.5%)	−1.0pp (−2.7 to 0.8)
Malignancy yield, excluding in situ	57/207 (27.5%)	60/415 (14.5%)	−13.1pp (−20.0 to −6.1)
Malignancy yield, including in situ	60/207 (29.0%)	63/415 (15.2%)	−13.8pp (−20.9 to −6.7)

## Discussion

This study compared a consultant-led FTF cohort (201 patients, 207 lesions) with a SAF teledermoscopy cohort (378 patients, 415 lesions), both assessed by the same dermoscopy-trained consultant over an identical six-month period within the ABUHB dermatology service. The most notable difference was in the discharge rate. Just over half of the teledermoscopy patients (206/378, 54.5%) were discharged without further intervention at their first assessment, compared with just over one-third of the FTF cohort (72/201, 35.8%; chi-square = 18.34, df = 1, p < 0.0001).

Much of this gap and the highly significant difference in the overall distribution of clinical outcomes (chi-square = 108.87, df = 5, p < 0.0001) is likely to reflect triage-related case selection rather than a true difference in diagnostic accuracy. Referrals with images that already appeared clinically concerning were sent directly to the FTF clinic, while less clinically concerning referrals were assessed through teledermoscopy. Therefore, the FTF cohort was, by design, weighted toward higher pre-triage suspicion from the outset. This pattern is also reflected in the cohorts' baseline demographics: the FTF group was significantly older (mean: 73.7 vs 59.6 years; Mann-Whitney U = 56,862, p < 0.0001) and had a higher proportion of male patients (108/201, 53.7% vs 156/378, 41.3%; χ²(1) = 8.21, p = 0.004). This may reflect the higher baseline risk profile of older male patients and the tendency for more concerning lesions to be triaged directly to FTF review. This is consistent with our earlier series, where teledermoscopy diverted between 75.6% of USC referrals and 83.0% of routine referrals away from the clinic across 1,203 cases [3], and with the high diversion rates reported elsewhere [10]. Seborrhoeic keratosis (116/415, 28.0%) and benign naevi (56/415, 13.5%) accounted for a significantly larger proportion of teledermoscopy diagnoses (chi-square = 10.03, p = 0.002 and chi-square = 19.16, p < 0.0001, respectively), again in line with our previous work [3] and with Abdul Gafoor et al.'s 91% diagnostic accuracy for seborrhoeic keratosis using patient-led teledermatology [11].

Among index lesions, malignancy yield followed a similar pattern to the discharge-rate difference above. Excluding in situ disease, yield was 57/207 (27.5%) in the FTF cohort compared with 60/415 (14.5%) in the teledermoscopy cohort (absolute difference: -13.1 percentage points, 95% CI: -20.0 to -6.1); including in situ disease, yield was 60/207 (29.0%) vs 63/415 (15.2%) (absolute difference: -13.8 percentage points, 95% CI: -20.9 to -6.7). As with the discharge-rate comparison, this most likely reflects triage-related case selection rather than a true difference in diagnostic performance: lower-risk referrals were routed to teledermoscopy by design, so a lower malignancy yield is expected and should not be interpreted as evidence of missed disease. BCC was the leading malignancy in both pathways (25/207 (12.1% FTF) vs 54/415 (13.0% teledermoscopy); absolute difference: 0.9 percentage points, 95% CI: -4.6 to 6.4), consistent with BCC being the most common skin cancer nationally and with our previous findings [3]. However, the distribution of other confirmed cancers differed between pathways: invasive SCC was concentrated in the FTF pathway (26/207 (12.6%) vs 4/415 (1.0%); absolute difference: -11.6 percentage points, 95% CI: -16.2 to -7.0), as was invasive melanoma to a lesser extent (6/207 (2.9%) vs 2/415 (0.5%); absolute difference: -2.4 percentage points, 95% CI: -4.8 to 0.0). In situ disease was rare and similarly distributed across both pathways (lentigo maligna: 0/207 vs 1/415; Bowen's disease: 3/207 (1.4%) vs 2/415 (0.5%)). Reporting these five categories separately, rather than combining melanoma with lentigo maligna and excluding Bowen's disease as originally presented, increased the overall malignancy yield by a similar small margin in both cohorts (+1.5 percentage points FTF, +0.7 percentage points teledermoscopy) and did not materially alter the between-cohort difference. This sensitivity analysis confirms our original endpoint definition did not meaningfully bias the headline comparison. All comparisons in this paragraph are descriptive and exploratory (see Methods); given the non-randomised triage design and small event counts for melanoma and in-situ disease, they should not be interpreted as head-to-head tests of diagnostic accuracy.

Importantly, malignancy was not confined to USC referrals. Within teledermoscopy, biopsy-proven BCC was identified across all referral groups and was the highest among routine referrals (15/67, 22.4%) compared with urgent (16/118, 13.6%) and USC (23/193, 11.9%) streams. These are modest denominators, so this comparison should not be over-interpreted. However, it highlights that diagnostic vigilance should be maintained regardless of referral priority. Referral onward to another specialty was similar across both pathways (6/201 (3.0% FTF) vs 10/378 (2.6% teledermoscopy)), which is reassuring, although this remains descriptive only. This suggests that teledermoscopy is not systematically failing to identify cases that require input from other specialties.

Incidental findings were a notable feature of the FTF cohort: 25 lesions across 201 consultations (12.4%), comprising 19 BCCs (76.0%), 4 SCCs (16.0%), 1 lentigo maligna (4.0%), and 1 clear cell acanthoma (4.0%). This means that 24 of the 25 incidental lesions were skin cancers. These lesions would not have been detected through teledermoscopy of the index lesion alone, since that pathway does not include total body skin examination (TBSE). Most were low-grade BCCs, but the lentigo maligna and, importantly, the four SCCs would all have warranted timely management. Therefore, while the incidental lesion burden here was dominated by low-risk lesions, it should not be dismissed as clinically negligible. This broadly mirrors Vestergaard et al.'s prospective work, which found incidental melanoma in 0.6% of FTF patients [12], and is consistent with Deda et al.'s point that the absence of TBSE is a genuine constraint of teledermatology [4]. The implication matters most for patients with elevated baseline risk, including those with a personal or family history of melanoma, multiple atypical naevi, previous non-melanoma skin cancer, or immunosuppression. In these patients, routing to FTF assessment regardless of the appearance of the index lesion may help address this limitation while preserving most of the teledermoscopy's efficiency gains for lower-risk patients.

Repeat photography was used in 16 teledermoscopy cases (4.2%), consistent with our earlier finding that patients monitored digitally are usually discharged once a naevus is confirmed to be benign [3]. We recommend that repeat photography be retained and, where feasible, formalised within teledermoscopy pathways as a structured monitoring option for lesions of uncertain significance, with clearly defined follow-up intervals and discharge criteria.

Taken together, these findings support the continued integration of teledermoscopy into NHS skin cancer referral pathways [2], in keeping with our earlier work [3,8,9]. The direct, same-service, same-consultant design strengthens this conclusion: teledermoscopy discharged substantially more patients than FTF assessment while still identifying skin cancer across referral categories. However, differences in discharge rate and malignancy yield between pathways should be interpreted as reflecting triage-related case selection and baseline case mix, rather than as a head-to-head comparison of diagnostic performance. The key limitations of teledermoscopy - lack of TBSE, dependence on image quality, and the need for careful patient selection - remain important, but appear manageable with appropriate governance, standardised imaging [4], and structured risk stratification for patients who require FTF assessment.

Limitations

Referrals were triaged by clinical images rather than randomised, so baseline risk differed between groups. Therefore, differences in discharge rate and malignancy yield are likely to reflect case mix and triage selection rather than true differences in diagnostic performance. The findings should therefore be interpreted as evidence of pathway throughput and service impact, rather than as a head-to-head comparison of diagnostic accuracy. The retrospective, single-service, single-consultant design over a six-month period may limit generalisability, although it reduces inter-clinician variability. Statistical analysis was applied to broad outcome distributions, diagnostic categories, and baseline cohort characteristics, but residual confounding remains likely. Finally, teledermoscopy is inherently limited by its inability to detect incidental lesions outside the photographed area. This is particularly relevant for higher-risk patients, who may still require FTF review. Long-term outcomes for discharged patients were not available, meaning that false-negative or missed-malignancy rates could not be assessed. Prospective follow-up studies are therefore needed to confirm pathway safety.

Referral urgency and clinical outcome were assigned per patient, while diagnostic and histological findings were assessed per lesion; this could introduce a modest degree of within-patient clustering for the 6 FTF and 37 teledermoscopy patients with more than one lesion, which was not formally modelled (e.g., with cluster-robust or mixed-effects methods). Given the small proportion of multi-lesion patients, we do not expect this to materially affect the reported associations, but it should be considered when interpreting lesion-level percentages alongside patient-level outcome data. Confirmation yields reported in Figure 3 and Figure 6 use the total number of clinically suspected lesions in each category as the denominator, including lesions not sent for biopsy or excision; these are therefore best interpreted as histological confirmation yields rather than diagnostic accuracy or positive predictive values and are subject to partial-verification bias, likely underestimating true clinical concordance. Yields for small subgroups, particularly lentigo maligna/melanoma in situ (n=1 in the teledermoscopy cohort), are based on very small numbers and should be interpreted with corresponding caution.

## Conclusions

Within the same dermatology service, time period, and assessing consultant, teledermoscopy was associated with a substantially higher discharge rate than FTF assessment while still identifying skin cancers across referral urgency categories. A meaningful part of this difference is likely attributable to upstream image-based triage, whereby lower-suspicion referrals were routed to teledermoscopy. These findings should therefore be understood as describing service throughput rather than as a head-to-head comparison of diagnostic performance between the two modalities.

Basal cell carcinoma was the leading confirmed malignancy in both pathways and was not confined to USC referrals, reinforcing the need for consistent diagnostic vigilance regardless of referral urgency. FTF assessment also identified a meaningful burden of incidental skin cancers, predominantly low-grade basal cell carcinomas, that would not have been detected through image-based referral assessment alone. Taken together, these findings support the continued integration of teledermoscopy into NHS skin cancer referral pathways, alongside structured risk stratification to ensure that patients with an elevated baseline risk of life-threatening or multiple skin cancers continue to receive FTF assessment and whole-body skin examination where appropriate.
